# Toll-like receptor 2 signaling is a mediator of apoptosis in herpes simplex virus-infected microglia

**DOI:** 10.1186/1742-2094-4-11

**Published:** 2007-04-30

**Authors:** Rajagopal N Aravalli, Shuxian Hu, James R Lokensgard

**Affiliations:** 1 Center for Infectious Diseases and Microbiology Translational Research, University of Minnesota Medical School, Minneapolis, MN 55455, USA

## Abstract

**Background:**

Information regarding the response of brain cells to infection with herpes simplex virus (HSV)-1 is needed for a complete understanding of viral neuropathogenesis. We have recently demonstrated that microglial cells respond to HSV infection by producing a number of proinflammatory cytokines and chemokines through a mechanism involving Toll-like receptor 2 (TLR2). Following this cytokine burst, microglial cells rapidly undergo cell death by apoptosis. We hypothesized that TLR2 signaling might mediate the cell death process as well.

**Methods:**

To test this hypothesis, we investigated HSV-induced cell death of microglia obtained from both wild-type and TLR2^-/- ^mice. Cell death was studied by oligonucleosomal ELISA and TUNEL staining, and the mechanisms of apoptosis were further analyzed using murine apoptosis-specific microarrays. The data obtained from microarray analysis were then validated using quantitative real-time PCR assays.

**Results:**

HSV infection induced apoptotic cell death in microglial cells from wild-type as well as TLR2 cells. However, the cell death at 24 h p.i. was markedly lower in knockout cells. Furthermore, microarray analyses clearly showed that the expression of pro-apoptotic genes was down-regulated at the time when wild-type cells were actively undergoing apoptosis, indicating a differential response to HSV in cells with or without TLR2.

**Conclusion:**

We demonstrate here that HSV induces an apoptotic response in microglial cells which is mediated through TLR2 signaling.

## Background

In the central nervous system (CNS), microglial cells generate the first line of defense against invading pathogens [1]. They are key immune cells that survey the brain parenchyma. During early onset of infection, microglia become activated and produce proinflammatory cytokines and chemokines. Production of these proinflammatory mediators may result in the infiltration of lymphocytes across the blood-brain barrier to sites of viral infection [2]. Microglia are functionally very similar to macrophages in that they clear up dead neurons and other cell debris by phagocytosis [1,2]. Therefore, efficient immune functions by microglial cells may be critical in controlling a number of CNS infections.

Herpes simplex virus 1 (HSV-1) is a neurotropic virus that infects a wide range of mammalian cells. Following primary infection of epithelial cells, HSV gains access to the nervous system and establishes latency in ganglionic neurons. Viral reactivation from this latent state may result in herpes encephalitis. A number of studies have demonstrated that Toll-like receptor (TLR) signaling in microglia is critical in generating innate immune responses against viral pathogens in the CNS [3-7]. In cell lines, HSV infection has been shown to activate signaling from TLR2 and TLR9 [4,8,9]. While TLR2 is localized on the cell surface, TLR9 is expressed intracellularly on lysosomal membranes. In a recent report, TLR2-deficient neonatal mice were found to be less susceptible to encephalitis caused by HSV, suggesting that TLR2 plays an important role in disease pathogenesis [4].

We have previously shown that microglial cells respond to HSV-1 by producing a large number of proinflammatory immune mediators in a TLR2-dependent manner [3]. Interestingly, however, these cells undergo apoptotic cell death following immune mediator production [10]. Although activation of TLR signaling has been shown to induce apoptosis in cell lines [11,12], little is known about TLR involvement in cell death of primary brain cells. In this study, we hypothesized that TLR2 signaling induces HSV-mediated microglial cell apoptosis.

## Methods

### Preparation of microglial cell cultures

Wild type and TLR2^-/- ^C57BL/6 mice were purchased from the Jackson Laboratories (Bar Harbor, ME). Purified microglial cell cultures (>99% pure), as determined by MAC-1 antibody staining (Roche Applied Science, Indianapolis, IN), were prepared from these mice using a previously described method with minor modifications [13]. Growth medium for microglial cell cultures was Dulbecco's modified Eagle's medium (DMEM) with 10% heat-inactivated fetal calf serum (HyClone Laboratories, Logan, UT) and antibiotics. For microarray analysis and real-time PCR assay, 1 × 10^6 ^cells/sample were used. For oligonucleosomal ELISA and TUNEL staining, 2 × 10^5 ^cells were used.

### Virus

A highly neurotropic HSV-1 17 syn^+ ^strain, propagated and purified from rabbit skin fibroblasts, was used for infection studies at a multiplicity of infection (MOI) of 2. After adding virus, culture plates were incubated at 37°C for the indicated time points.

### Oligonucleosomal ELISA

A sandwich ELISA-based system (Roche Applied Science) was used to detect nucleosomes generated due to DNA fragmentation during apoptosis. The assay was performed at the indicated time points as per the manufacturer's instructions. Data are representative of three independent experiments, and bars represent the mean + SD of triplicate samples.

### TUNEL staining

Wild type and TLR2^-/- ^microglial cells were infected with HSV (MOI = 2) and DNA fragmentation was determined by terminal deoxynucleotidyl transferase (TdT)-mediated dUTP-X nick end labeling (TUNEL) using the ApopTag ^® ^peroxidase*in situ *apoptosis detection kit (Millipore, Temecula, CA). Microglial cells were cultured on Lab-Tek chamber slides at a density of 2 × 10^5 ^cells per well. At the end of the incubation period, cells were fixed in 4% paraformaldehyde for 20 min followed by a staining procedure according to the manufacturer's protocol.

### Microarrays

Mouse-specific OligoGEArray^® ^apoptosis microarrays (OMM-12) (SuperArray, Frederick, MD) were used for our studies, and hybridization procedures were performed per manufacturer's instructions. Wild type and TLR2^-/- ^microglial cells were treated with HSV, and total RNA was extracted after 8 h and 24 h post-infection (p.i.) using the RNeasy mini kit (Qiagen, Valencia, CA). Following the chemiluminescent detection steps, positive spots on arrays were scanned using a Kodak Image Station 2000R (Molecular Imaging Systems, Rochester, NY) and were quantified using GEA analysis suite software (SuperArray). Data were analyzed as relative induction after each gene was normalized to the house-keeping gene GAPDH.

### Quantitative real-time PCR

cDNA was synthesized using 1 μg of total RNA from uninfected and infected wild-type and TLR2^-/- ^microglia, at 8 h and 16 h p.i,. using Superscript II reverse transcriptase (Invitrogen, Carlsbad, CA) and oligo dT_6–12 _primers (Sigma-Genosys, The Woodlands, TX). PCR was performed with the FullVelocity SYBR Green QPCR master mix (Stratagene, La Jolla, CA). The PCR conditions for the Mx3000P QPCR System (Stratagene) were: 40 denaturation cycles of 95°C for 10 s, annealing at 60°C for 10 s and elongation at 72°C for 10 s. The relative product levels were quantified using the 2(-Delta Delta C(T)) method [14] and were normalized to β-actin, and are representative of three independent experiments. Forward and reverse primer sequences used in the study: caspase-3: 5'-gggcctgaaataccaagtca-3' and 5'-aaatgaccccttcatcacca-3'; Dsip1: 5'-ggtggccctagacaacaaga-3' and 5'-tcaagcagctcacgaatctg-3'; CIDE-B: 5' ctggaactcagctcctccac-3' and 5'- cctccaggaccagtgttagc-3'; caspase-2: 5'- cagctccaagaggtttttcg-3' and 5'- acatccaggggattgtgtgt-3'; Tnfrsf12a: 5'-gattcggcttggtgttgatg-3' and 5'-cagtccatgcacttgtcgag-3'; RipK2: 5' cagctgggatggtatcgttt-3' and 5'- tggttaaggcaggcttcagt-3'.

## Results and discussion

### TLR2 signaling mediates HSV-induced apoptosis in murine microglia

**Figure 1:**
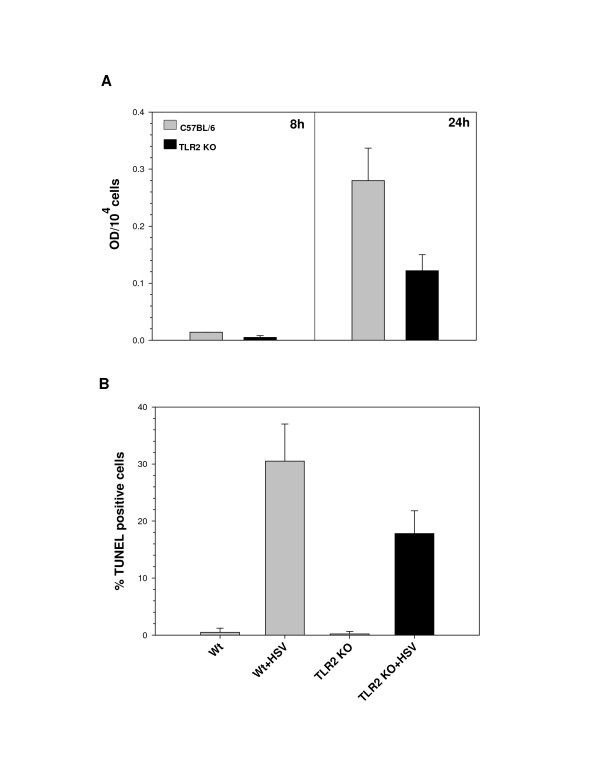
HSV infection induces apoptosis in murine microglial cells. Wild-type and TLR2^-/- ^C57BL/6 microglial cells were infected with HSV at a MOI of 2. (A) The cells were examined for apoptotic DNA fragmentation using an oligonucleosomal ELISA at 8 and 24 h p.i. Data are presented as optical density (OD) per 10^4 ^cells and are representative of three independent experiments using cells isolated from different brain specimens. (B) TUNEL staining of wild-type and TLR2^-/- ^microglia at 24 h p.i. After fixing and staining the wells, TUNEL positive cells from at least five fields were counted for each well. Data presented were representative of three independent experiments.

To test the hypothesis that TLR2 signaling is involved in the induction of apoptosis in HSV-infected microglia, 2 × 10^5 ^cells/sample were infected with the neurotropic HSV-1 strain 17 syn^+^. We have previously demonstrated that HSV infects both wild-type and TLR2^-/- ^microglia with similar efficiencies [3], and that apoptosis in virus-infected wild-type microglia peaks at 24 h p.i. [10]. Following these observations, we harvested cells at 8 h and 24 h p.i., to reflect early and peak apoptotic time points, and performed oligonucleosomal ELISA assay to detect nucleosomes generated as a result of DNA fragmentation. As shown in Fig. 1A, cell death was not observed at significant levels in either wild-type or TLR2^-/- ^microglia at 8 h p.i. However, apoptosis was induced in wild-type cells at 24 h p.i. The extent of HSV-induced cell death observed in TLR2^-/- ^microglia at 24 h p.i. was 40% of that seen in wild-type microglia. To confirm apoptotic death in these cells, TUNEL assay was performed using wild type and TLR2^-/- ^microglial cells following a 24 h infection with HSV. As shown in Fig. 1B, HSV-induced apoptosis was found to be markedly lower in TLR2^-/- ^microglia than in wild type cells, further demonstrating that TLR2 signaling plays a role in regulating microglial cell apoptosis in response to HSV.

### Differential expression of apoptotic genes in HSV-infected wild-type and TLR2^-/- ^microglial cells

To further investigate differences in cell death between HSV-infected wild-type and TLR2^-/- ^cells, and to study the expression profiles of apoptotic genes, we performed microarray analyses using mouse-specific apoptosis microarrays. These arrays contained most murine apoptotic genes. Since gene expression occurs several hours ahead of DNA fragmentation, 8 h and 16 h p.i. time points were selected for this study. Furthermore, an induction or down-regulation of at least two-fold or higher of a given gene was considered significant in either inducing or blocking apoptosis. As shown in Tables 1 and 2, the expression profiles of the apoptotic genes were markedly different between wild-type and in TLR2^-/- ^microglial cells. At 8 h p.i., the expression of most apoptotic genes remained unchanged in wild-type cells, while TLR2^-/- ^cells showed induction of few genes. At 16 h p.i., however, pro-apoptotic genes such as caspase-3 and caspase-8 were highly expressed in wild-type cells demonstrating that they were actively undergoing apoptosis. Interestingly, these genes were not expressed in TLR2^-/- ^cells at this time point. Moreover, many pro-apoptotic genes were down-regulated in TLR2^-/- ^cells at 16 h p.i. when compared with their expression at 8 h p.i.

**Table 1:** Expression of apoptotic genes in HSV-infected microglial cells from C57BL/6 mice

Symbol	Gene	Fold change	
		8 h	16 h
Casp3	Caspase-3	2.39	3.79
Card15	Caspase recruitment domain family member 15	-	2.04
Casp11	Caspase-11	0.85	2.38
Casp8	Caspase-8	-0.67	2.41
Dsip1	TSC22 domain family 3	-0.26	3.04
Tnfrsf12a	TNF receptor superfamily member 12a	2.32	0.84
Atf5	Activating transcription factor	-2.24	-1.22
Bcl10	B-cell leukemia/lymphoma 10	-2.50	-0.94
Bid	BH3 interacting domain death agonist	-2.15	-1.03
Dad1	Defender against cell death	-2.17	-1.40
Mapk8ip1	MAP kinase interacting protein 1	-4.02	-4.06
Rnf7	Ring finger protein 7	-4.44	-1.81
Polb	Polymerase B	-5.08	-1.82
Prdx2	Peroxiredoxin 2	-3.49	-2.14
Ltbr	Lymphotoxin B receptor	-3.00	-1.98
Tnfrsf21	TNF receptor superfamily member 21	-4.70	-5.88
Traf3	TNF receptor-associated factor 3	-3.52	-2.07

**Table 2:** Expression of apoptotic genes in HSV-infected TLR2KO microglial cells.

Symbol	Gene	Fold change	
		8 h	16 h
Tnfsf10	TNF ligand superfamily member 10	8.66	5.59
Casp12	Caspase-12	4.15	1.00
RipK2	Receptor (Tnfrsf)-interacting kinase2	3.38	2.87
Casp8ap2	Caspase-8-associated protein 2	3.36	0.66
Fasl	Fas ligand	3.15	1.37
Casp3	Caspase-3	2.91	1.46
Tnf	Tumor necrosis factor	2.71	1.35
Cflar	Casp-8 and FADD-like apoptosis regulator	2.50	0.90
Tnfrsf5	TNF superfamily member 5	2.35	0.93
Bag4	BCL2-associated athanogene 4	2.16	0.58
Bad	Bcl-associated death promoter	-3.00	-5.07
Akt	Thymoma viral proto-oncogene1	-3.76	-2.56
Als2cr2	Als chromosome region candidate 2	-3.97	-2.38
Bax	Bcl2-associated X protein	-2.35	-1.49
Bcl10	B-cell leukemia/lymphoma 10	-2.71	-3.44
Birc5	Baculoviral IAP repeat-containing 5	-2.19	-8.87
Bcl2l14	Bcl2-like 14 (apoptosis facilitator)	-2.08	-4.08
Bid	BH3 interacting domain death agonist	-0.97	-2.21
Bnip3l	BCL2/adenovirus E1B-interacting protein	-4.94	-5.14
Birc6	Baculoviral IAP repeat-containing 6	-1.35	-3.19
Bnip2	BCL2/adenovirus E1B-interacting protein	-1.04	-2.56
Api5	Apoptosis inhibitor 5	-4.01	-2.56
Dsip1	TSC22 domain family 3	-2.55	-1.50
Cideb	Cell-death inducing DNA fragmentation factor 2	-1.78	-3.42
Cradd	CASP2 and RIPK1 adaptor domain containing protein	-1.54	-6.32
Fadd	Fas-associated death domain	-1.21	-2.92
Faim	Fas apoptotic inhibitory molecule	-1.81	-3.18
Hells	Helicase lymphoid specific	-0.84	-2.24
Il10	Interleukin 10	-1.27	-2.21
Mapk8ip1	MAP kinase interacting protein 1	-0.87	-5.75
Zc3hc1	C3HC type zinc finger protein	-0.93	-2.25
Nfkb1	NF-κB	-1.92	-1.55
Rnf7	Ring finger protein 7	-2.81	-2.31
Pak7	P21 (CDKN1A)-activated kinase 7	-0.85	-2.03
Traf3	TNF receptor-associated factor 3	-6.31	-2.71
Tnfrsf21	TNF receptor superfamily member 21	-1.87	-1.75
Trp53	P53	-2.80	-1.17

### Validation of apoptotic gene expression in HSV-infected microglia

**Figure 2:**
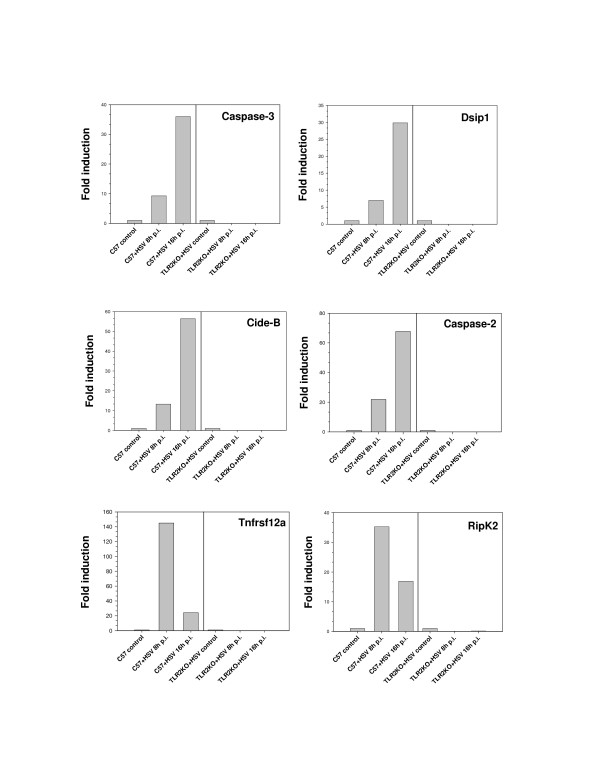
Differential expression of apoptotic genes in microglial cells obtained from wild-type and TLR2^-/- ^mice. Real-time PCR was performed using RNA from uninfected and HSV-infected microglia with primers specific for the apoptotic genes indicated. β-actin was used to normalize the values of apoptotic genes tested. Data presented are representative of three independent experiments.

To further confirm these findings, we performed quantitative real-time PCR for six different apoptotic genes selected from the microarray data. These genes were found to be either up-regulated or down-regulated in HSV-infected wild-type microglia and were down-regulated in TLR2^-/- ^cells (Tables 1 &2). As shown in Fig. 2, the expression levels of caspase-2, caspase-3, Cide-B and Dsip1 increased in wild-type cells between 8 h to 16 h p.i. whereas they were down-regulated from basal expression in uninfected controls. On the other hand, Tnfrsf12a and RipK2 were down-regulated both in wild-type and TLR2^-/- ^cells. These data further demonstrate that TLR2^-/- ^cells have significantly lower levels of pro-apoptotic gene expression than wild-type cells, and that TLR2 signaling mediates apoptotic cell death in HSV-infected microglial cells. We have previously shown that the levels of TNF-α expression in TLR2^-/- ^microglia were approximately 50% of those seen in wild-type cells [3]. In this study, apoptotic death in TLR2^-/- ^cells was found to be 40% of that in wild-type microglia and, therefore, it is possible that TNF-α, as well as other immune mediators, might eventually trigger apoptosis in cells lacking TLR2.

## Conclusion

In this study, we showed that TLR2 signaling induces apoptosis in HSV-infected microglia. Although the virus infects both wild-type and TLR2^-/- ^microglial cells with similar efficiencies [3], apoptotic cell death was significantly lower in TLR2^-/- ^cells. In addition, a large number of pro-apoptotic genes were clearly down-regulated in TLR2^-/- ^cells at a time when wild-type cells were actively undergoing apoptosis. We have previously demonstrated that at early time points the production of proinflammatory immune mediators did not occur in TLR2^-/- ^microglia but they were produced robustly in wild-type cells [3]. However, TNF-α was still expressed in TLR2^-/- ^cells at approximately 50% of the level seen in wild-type cells, and it is possible that immune mediators such as TNF-α produced early in infection, might induce apoptosis. In a recent study, we deduced apoptotic pathways occurring in primary glial cells infected with HSV and found that TNF-α pathway was active in HSV-infected microglial cells [10]. Taken together, these data indicate that HSV infection of microglial cells activates TLR2 signaling which, in turn, induces the production of immune mediators and eventually leads to cell death.

## Competing interests

The author(s) declare that they have no competing interests.

## Authors' contributions

RNA and JRL conceived the study and its design, and analyzed the data. RNA and SH performed the experiments. RNA drafted the manuscript. All authors read and approved the final manuscript.
